# Deuterium Isotope Effects on ^13^C-NMR Chemical Shifts of 10-Hydroxybenzo[*h*]quinolines

**DOI:** 10.3390/molecules18044544

**Published:** 2013-04-17

**Authors:** Poul Erik Hansen, Fadhil S. Kamounah, Daniel T. Gryko

**Affiliations:** 1 Department of Science, Systems and Models, Roskilde University, P.O. Box 260, DK-4000 Roskilde, Denmark; E-Mail: fadhil@ruc.dk; 2 Institute of Organic Chemistry, Polish Academy of Sciences, Kasprzaka 44/52, 01-224 Warsaw, Poland; E-Mail: danieltgryko@gmail.com

**Keywords:** 10-hydroxybenzo[*h*]quinolones, deuterium isotope effects on ^13^C chemical shifts, intramolecular hydrogen bonds, hydrogen bond potentials, DFT calculations

## Abstract

Deuterium isotope effects on ^13^C-NMR chemical shifts are investigated in a series of 10-hydroxybenzo[*h*]quinolines (HBQ’s) The OH proton is deuteriated. The isotope effects on ^13^C chemical shifts in these hydrogen bonded systems are rather unusual. The formal four-bond effects are found to be negative, indicating transmission via the hydrogen bond. In addition unusual long-range effects are seen. Structures, NMR chemical shifts and changes in nuclear shieldings upon deuteriation are calculated using DFT methods. Two-bond deuterium isotope effects on ^13^C chemical shifts are correlated with calculated OH stretching frequencies. Isotope effects on chemical shifts are calculated for systems with OH exchanged by OD. Hydrogen bond potentials are discussed. New and more soluble nitro derivatives are synthesized.

## 1. Introduction

10-Hydroxybenzo[*h*]quinolines (HBQs) display excited-state intermolecular proton transfer (ESIPT), which results in interesting photophysical properties [1,2,3,4,5,6,7,8,9,10]. ESIPT is, among others, a direct consequence of very strong intramolecular hydrogen bonding [2,3], and it has been recently studied both experimentally and theoretically. Needless to say, the strength of hydrogen bonds between the basic nitrogen atom and the OH group, has direct consequences on the photophysical properties of these molecules. The distance between the two heavy atoms of the hydrogen bond is relatively short and a potentially strong hydrogen bond thus results. Therefore, this type of molecule provides an interesting opportunity for exploring methods and parameters describing intramolecular hydrogen bonds and in addition, for getting information about the hydrogen bond potentials. In addition to heavy atom distances, all bond lengths and distances of the hydrogen bond system can also be useful parameters [11]. OH chemical shifts and two-bond isotope effects on ^13^C-NMR chemical shifts, ^2^ΔC(OD) [12,13,14], have been used to characterize hydrogen bond systems. Deuterium isotope effects can be observed not only over two bonds, but also long range [15,16]. An interesting possibility is the transmission of the effects via hydrogen bonds [11]. Recently, a study of calculations of OH stretching frequencies in the B3LYP/6-31G(d) approach showed a very good correlation between the OH stretching frequency and ^2^ΔC(OD), the acceptor being oxygen [17,18].

HBQs seems a very good group to study some of the abovementioned phenomena and also to establish whether these compounds are tautomeric or not, considering that 12-hydroxy-1-azaperylene, a close analogue, has been found to be tautomeric [19,20,21].

**Figure 1:**
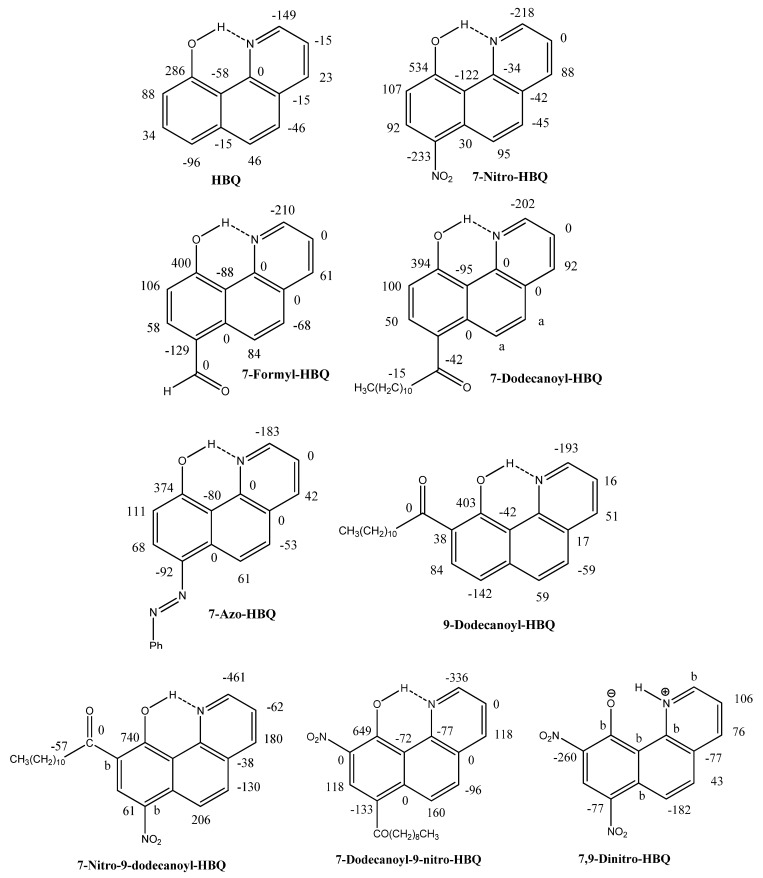
Deuterium isotope effects on ^13^C-NMR chemical shifts of HBQs in ppb.

In order to achieve some of the above mentioned goals it has been necessary to synthesize new compounds with strong hydrogen bonds, that are soluble in solvents such as CDCl_3_ or CD_2_Cl_2_ (see Figure 1).

### 2.1.Assignments

The ^1^H-NMR spectrum of HBQ can be assigned based on chemical shifts and spin-system patterns. For the H-7, H-8 and H-9 spin system, the latter is known to be at the lowest frequency due to the OH substituent. For the H-2, H-3 and H-4 spin system the first can be assigned the resonance to the highest frequency, in analogy with pyridine and quinoline. The coupling constants of the two spin systems are so different that an unambiguous assignment can be made. For the 7,9-dinitroderivative a coupling is seen between the XH proton and H-2. The coupling is larger in DMF-*d_7_* than in tetrahydrofuran-*d_8_*:CDCl_3_ (2:1). This coupling disappears upon addition of D_2_O. In the ^13^C-NMR spectrum of 7,9-dinitro-HBQ eleven sharp resonances can be clearly observed. In addition, two broad resonances are found.

Based on the ^1^H- chemical shifts, ^13^C-NMR chemical shifts of HBQ can be assigned based on HMQC and HMBC spectra. For ^13^C data see Table 1. The chemical shifts of the substituted compounds are assigned based on the data for HBQ and substituent effects, as most of the compounds have rather low solubility. Substitution at the 7-position can be seen very clearly as a high frequency shift of the H-6 proton. In addition, ^3^*J*(H-5,H-6) > ^3^*J*(H-7,H-8) > ^3^*J*(H-8,H-9). In addition, isotope effects may be useful. An example is distinction between substitution at the 7- or 9-position for dodecanoyl derivatives. The observation of a small deuterium isotope effect at the aliphatic C-2' carbon in the 7-nitro-9-dodecanoyl derivative ensures the assignment of this isomer. The experimental chemical shifts are also compared to calculated nuclear shieldings (see later).

**Table 1:** ^13^C-NMR chemical shifts of HBQ and HBQ derivatives in CDCl_3_ in ppm relative to TMS.

Compounds	HBQ	4-mor-pho-lino-HBQ	4-tosyl-HBQ ^a^	7-formyl-HBQ	7-azo-HBQ ^b^	7-nitro-HBQ ^c^	7-dode-canoyl-HBQ	9-dode-canoyl-HBQ	7-dode-canoyl-9-nitro-HBQ	7-nitro-9-dode-canoyl-HBQ	7,9-dinitro-HBQ (NH-form)
C-2	144.8	145.5	144.7	145.5	145.4	147.8	145.0	145.0	145.6	145.3	131.9 ^d^
C-3	120.7	109.4	120.0	121.7	121.3	124.0	121.4	121.2	122.5	122.5	124.6
C-4	136.0	156.9	149.9	136.9	136.6	138.9	132.9	136.9	138.0	138	145.7
C-4a	126.1	119.9	121.7	126.1	126.4	126.7	126.7	126.5	126.1	126.4	127.3 ^d^
C-5	124.6	120.3	119.2	128.1	124.4	130.3	126.0	126.8	123.9	128.7	132.5 ^e^
C-6	128.9	129.8	131.9	125.4	125.6	130.2	127.0	129.0	130.0	127.0	127.9 ^e^
C-6a	134.9	134.8	134.4	134.4	134.1	129.2	133.9	137.8	137.3	132.7	134.3
C-7	118.0	117.7	118.6	122.4	139.8	138.0	125.8	117.8	116.1	136.4	132.2 ^f^
C-8	129.8	127.6	131.0	140.4	117.3	123.5	136.4	130.5	130.0	129.8	125.2
C-9	113.8	113.8	115.2	113.8	114.7	113.9	112.8	123.4	130.4	124.3	143.3
C-10	159.3	159.6	159.1	165.8	165.4	166.4	163.5	161.2	166.1	160.6	167.2 ^d^
C-10a	115.8	116.6	115.5	115.6	115.2	115.5	116.0	116.2	122.3	117.2	112 ^f^
C-10b	148.1	149.4	145.2	147.4	148.1	147.2	147.7	148.6	147.2	147.5	144.3 ^d^
C=O	-	-	-	192.1			203.2	202.6	200.2	201.9	-

^a^ Benzene resonances: 137.1; 130.2; 128.0; 145.3; ^b^ Benzene resonances: 153.3; 122.8; 130.3; 129.1; ^c^ Solvent DMF-*d_7_*; ^d^ Observed in sample without D_2_O; ^e^ May be interchanged; ^f^ Very broad. Only seen in samples with excess D_2_O.

### 2.2. Deuterium Isotope Effects

2.2.1. ^13^C-NMR Chemical Shifts

**Figure 2:**
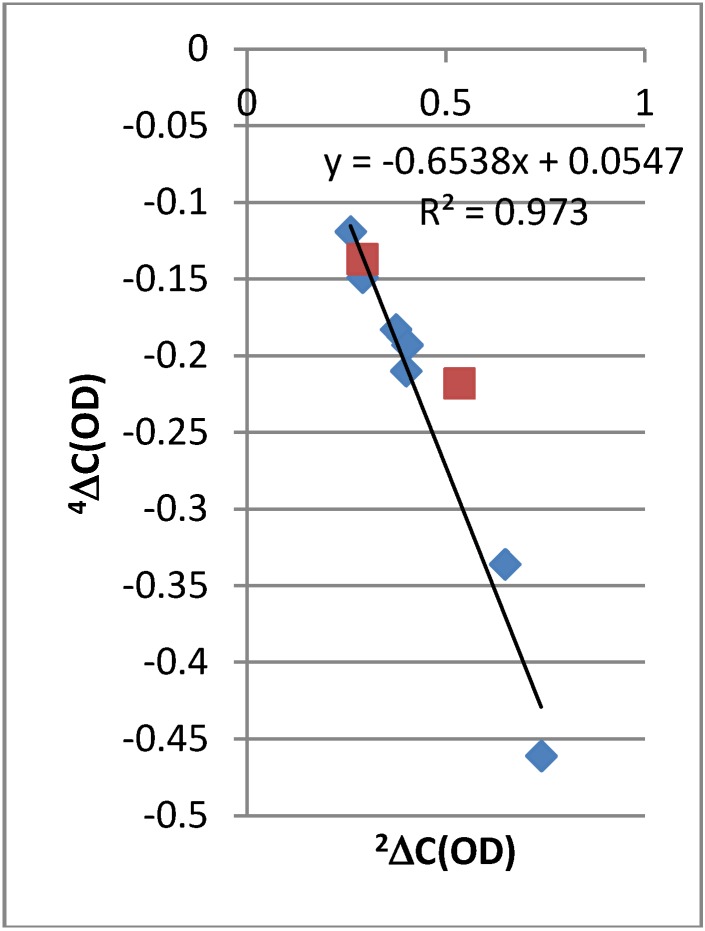
Plot of four-bond deuterium isotope effects on ^13^C chemical shifts *vs.* two-bond deuterium isotope effects on ^13^C chemical shifts. Red squares are data for HBQ and 4-nitro-HBQ in DMF-*d_7_*.

The isotope effects on chemical shifts are defined as ^n^ΔC(OD) = δC(OH) − δC(OD). The deuterium isotope effects on ^13^C chemical shifts in the compounds are given in Figure 1. ^2^ΔC(OD) of the 7-nitro-9-dodecanoyl-HBQ derivative is the largest deuterium isotope effect on carbon chemical shifts effect observed in a non-tautomeric system. It is remarkable that so many long-range effects are seen in these non-tautomeric systems. A rough correlation is found between ^2^ΔC(OD) and ^4^ΔC(OD) (Figure 2). It is interesting to notice that the data point for the 4-nitro-HBQ recorded in DMF-*d_7_* falls off the correlation line.

2.2.2. ^1^H-NMR Chemical Shifts

Deuterium isotope effects on ^1^H chemical shifts are seen in both the 9-dodecanoyl- (very small effects), 7-nitro-9-dodecanoyl- and 7-dodecanoyl-9-nitro-HBQ. The effects are as large as 32 ppb.

### 2.3. Chemical Shifts

#### 2.3.1. Solvent Effects

The ^1^H-NMR chemical shifts of the OH groups are rather high and especially that of the dinitro derivative is unusually high—20.2 ppm. The chemical shifts depend to some extent on the solvent. In DMF-*d_7_* the ^1^H chemical shifts are ~0.4 ppm higher than in CDCl_3_ (Table 2), exceptions are H-5 and H-8. For the OH signals this effect is 0.9 ppm in the 7,9-dinitro-HBQ (see later).

**Table 2:** .^1^H-NMR chemical shifts in ppm. Solvent CDCl_3_.

Comp.	HBQ	7-Dodecanoyl-HBQ	7-Formyl-HBQ	7-Nitro-HBQ ^a^	7-Azo-HBQ	9-Dode-canoyl-HBQ	9-Azo-HBQ	7-Nitro-9-dode-canoyl-HBQ	7-Dodecanoyl-9-nitro-HBQ	7,9-Dintro-HBQ ^b^
H-2	8.80 (9.02) ^c^	8.80	8.87	9.13	8.84	8.86	8.80	8.92	8.95	9.11 (9.47)
H-3	7.52 (7.81)	7.59	7.65	7.98	7.61	7.62	7.38	7.80	7.80	7.98 (8.31)
H-4	8.21 (8.61)	8.26	8.35	8.75	8.31	8.32	8.28	8.50	8.48	8.77 (9.23)
H-5	7.51 (7.90)	7.55	7.87	8.22	7.79	7.73	7.65	8.01	8.01	8.27 (8.58)
H-6	7.97 (7.98)	8.95	9.43	8.55	9.10	7.80	7.74	9.05	8.80	8.92 (9.01)
H-7	7.39 (7.55)	-	-	-	-	7.38	^d^	-	-	-
H-8	7.61 (7.69)	8.11	8.09	8.68	8.22	8.13	^d^	8.87	8.95	9.18 (9.27)
H-9	7.24 (7.21)	7.19	7.30	7.25	7.29	-	-	-	-	-
OH	14.90 (14.80)	16.11	16.40	16.68	15.74	16.39	15.69	19.11	18.21	19.45 (20.2)

^a^ Solvent DMF-*d_7_*. For data in CDCl_3_ see Ref. [22]; ^c^ Solvent THF-d_8_:CDCl_3_: (2:1); ^c^ Values in parentheses, solvent DMF-*d_7_*. ^d^ Overlapping lines.

#### 2.3.2. Ring Current Effects

**Figure 3:**
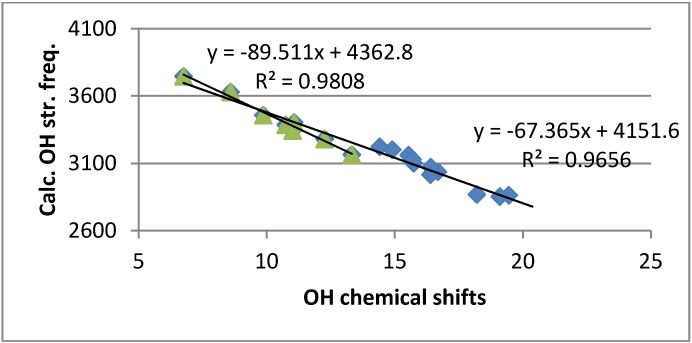
Plot of calculated OH stretching frequencies *vs.* OH chemical shifts ^a^.

^a^ The green data set is simple benzene derivatives. Data taken from Ref. [17]. The blue data are 10-hydroxybenzoquinolines from the present study.

**Figure 4:**
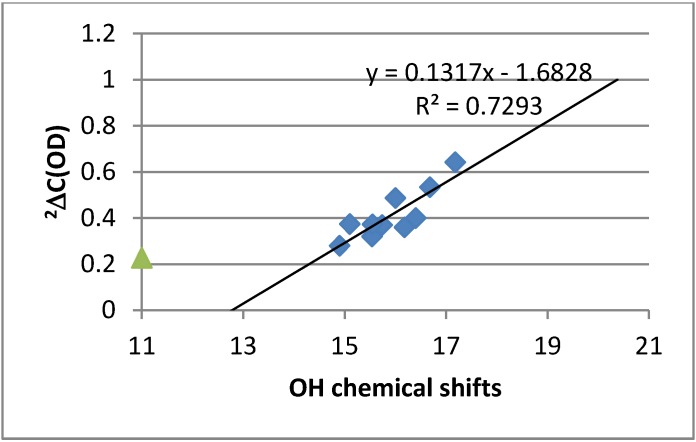
Plot of ^2^ΔC(OD) *vs.* OH chemical shifts. Green triangle is data for salicylaldehyde.

Ring current effects can be rather large in polycylic aromatic systems [23]. From Figure 3 it can be seen that the OH chemical shifts correlate with calculated OH stretching frequencies. However, the slopes are different for simple benzene derivatives and for 10-hydroxybenzoquinolines. This can be ascribed to differences in ring currents. For HBQ the effect can be estimated to be 1.1 ppm larger than for the benzene derivatives making a comparison with data for salicylaldehyde (see Figure 4).

### 2.4. Calculations

#### 2.4.1. Structures

The structures of HBQ and derivatives are calculated using DFT methods in the B3LYP/6-31G(d) scheme. The OH bonds are remarkably short [0.9968 Å (HBQ) to 1.0144 Å for the 7,9-dinitro-HBQ]. For both the 7-nitro- and the 7,9-dinitro-HBQ the calculations show a 20° twist of the nitro groups. For the 7- and the 9-azo-HBQ’s different rotamers have been explored. The same is true for the dodecanoyl derivatives. For the dodecanoyl derivatives the long chain is mimicked by use of an ethyl group. For the 7-dodecanoyl-HBQ the twist angle is as low as 8°. For the 9-dodecanoyl-HBQ the one with the C=O group twist out of the plane and pointing away from the OH groups has the lowest energy. However, fits between calculated ^1^H and ^13^C nuclear shieldings and ^1^H and ^13^C chemical shifts, respectively, points towards the conformer with the C=O group twisted out of the plane but pointing towards the OH group. The one in which the C=O groups forms a hydrogen bond to the OH group give a higher energy. The structures shown in Figure 1 are the ones best reproducing chemical shifts and isotope effects. It is seen that the O…N distance and the OH bond lengths correlate very well as also found earlier in other intramolecularly hydrogen bonded systems [11].

#### 2.4.2. Chemical Shifts

**Figure 5:**
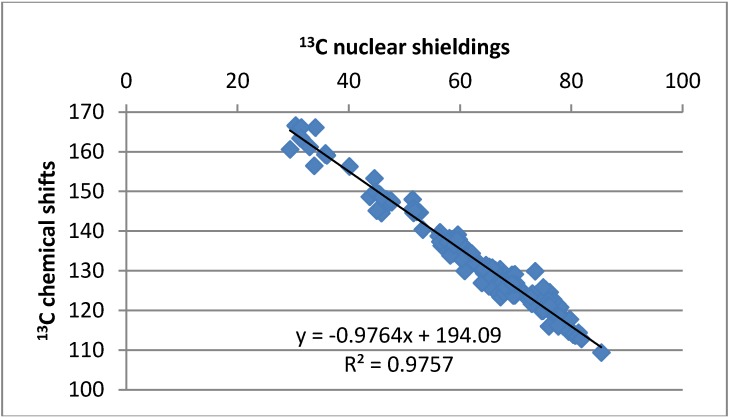
Plot of ^13^C-NMR chemical shifts *vs.* calculated ^13^C nuclear shieldings.

**Figure 6:**
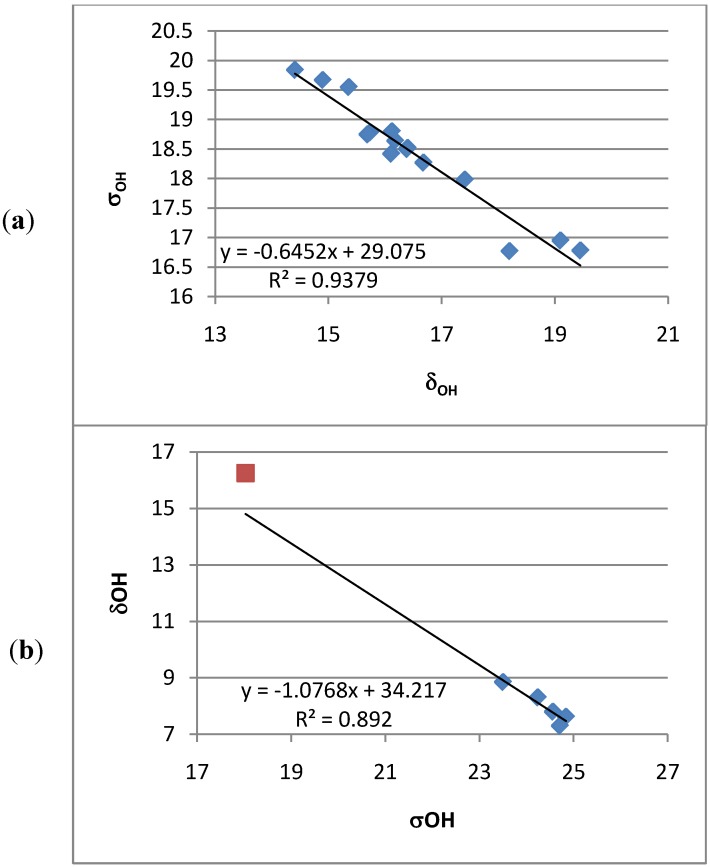
(**a**) Plot of OH nuclear shieldings *vs.* OH chemical shifts. (**b**) Plot of ^1^H nuclear shieldings *vs.* ^1^H chemical shifts for 9-dodecanoyl-HBQ.

^13^C-NMR chemical shifts were calculated. A reasonable correlation is found except for the carbons carrying protons and being either ortho or peri to nitro groups (Figure 5). Likewise, OH chemical shifts are calculated and the calculated nuclear shieldings show a reasonable correlation with OH chemical shifts. However, the slope of the line is very far from one (Figure 6). For the 7,9-dinitro derivative the experimental chemical shift used is that observed in THF-d_8_:CDCl_3_ (2:1).

#### 2.4.3. Calculations of Changes in Nuclear Shieldings

The changes are calculated for a OH bond length corresponding to the optimized structure and one with a OH bond length 0.01 Å shorter to mimic deuteriation. In the Jameson approach [24] [see Equation (1)] the isotope effect is given as the difference in the chemical shielding for the light and heavier isotope (marked with *):

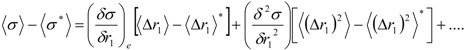
(1)
where <Δr>^2^ is the mean square amplitude.

**Figure 7:**
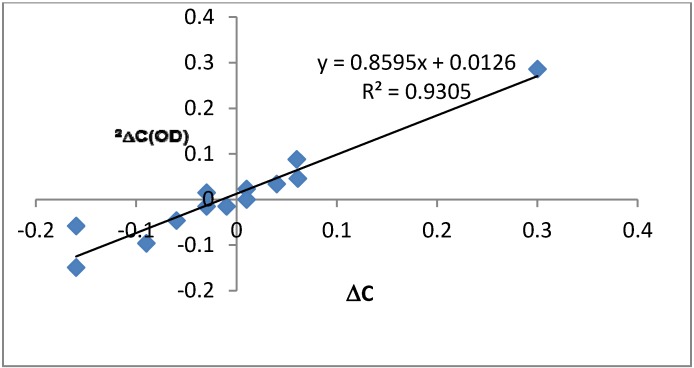
Plot of two-bond deuterium isotope effects on ^13^C chemical shifts *vs.* the change in the nuclear shielding ^a^ for HBQ.

^a^ The change in the OH bond lengths was set to 0.01 Å.

From equation (1) it is seen that the isotope effects on nuclear shielding depend primarily on the change in the nuclear shielding caused by a change of the OH bond length (first term in the product). This is an important part as it gives the sign of the isotope effect. The other factor (in square brackets) is the change in the OH bond length as a consequence of deuteriation. This part will depend on the shape of the hydrogen bond potential. The change in the nuclear shieldings for carbon x with the deuterium at nitrogen n bonds away (called ^n^ΔσC-x(OD) are calculated and given in Table 3 . Changes in nuclear shielding are calculated for HBQ, the 7-formyl-, the 7-dodecanoyl-, the 7-nitro-, the 7-dodecanoyl-9 nitro- and the 7-nitro-9-dodecanoyl-HBQ derivatives as well as for the 9-dodecanoyl hydrogen bonded to the C=O group and for the NH-form of 7,9-dinitro-HBQ. A number of trends can be seen, ^2^ΔσC-10(OD) is increasing with nitro-group substitution and ^4^ΔσC-6a(OD) change sign as a function of nitrogen substitution. Furthermore, ^4^ΔσC-2(OD) is clearly negative. These trends confirm what is found for isotope effects in the experimental spectra although the numbers are not identical as the change in the OH bond length upon deuteriation is set arbitrarily to a fixed value of 0.01 Å for all compounds. However, the variation due to substitution is rather small. The larger variation comes as a function of the change in the OH bond length upon deuteriation. This can be judged from plots of changes in nuclear shieldings *vs.* observed isotope effects. A plot for HBQ is seen in Figure 7. Slopes are found to be HBQ (0.80), 7-dodecanoyl- (1.23), 7-formyl- (1.32), 7-nitro- (1.44), 7-dodecanoyl-9-nitro- (1.87) and 7-nitro-9-dodecanoyl-HBQ (2.33). For the OH nuclear shieldings the value is ~0.45 ppm for all compounds.

**Table 3:** Changes in ^13^C nuclear shieldings due to OH bond length ^a^ changes ppb/0.01Å.

Compounds	HBQ	7-Dodecanoyl-HBQ	7-Carb-aldehyde-HBQ	7-Nitro-HBQ	7-Nitro-9-dodecanoyl-HBQ	9-Dodecanoyl-HBQ ^b^	7,9-Dinitro-HBQ (NH-form)
C-2	−160	−160	−170	−160	−188	20	190
C-3	−30	−30	−20	−10	−31	−10	130
C-4	10	30	30	35	39	−10	−60
C-4a	−10	−20	−30	−10	−28	−20	-80
C-5	−60	−70	−70	−60	−76	20	40
C-6	60	90	70	110	102	0	−90
C-6a	−30	−10	−20	30	0	50	−130
C-7	−90	−100	−90	−150	−128	−80	70
C-8	40	50	20	60	77	−30	−40
C-9	60	30	40	30	41	−110	−140
C-10	300	300	290	330	321	380	−20
C-10a	−160	−150	−140	−150	−139	50	−70
C-10b	10	−0	−20	−10	−38	0	200

^a^ For the 7,9-dintro it is the NH-form and the NH bond length is changed. ^b^ Rotamer with OH hydrogen bond to C=O group.

#### 2.4.4. Calculation of Frequencies

**Figure 8:**
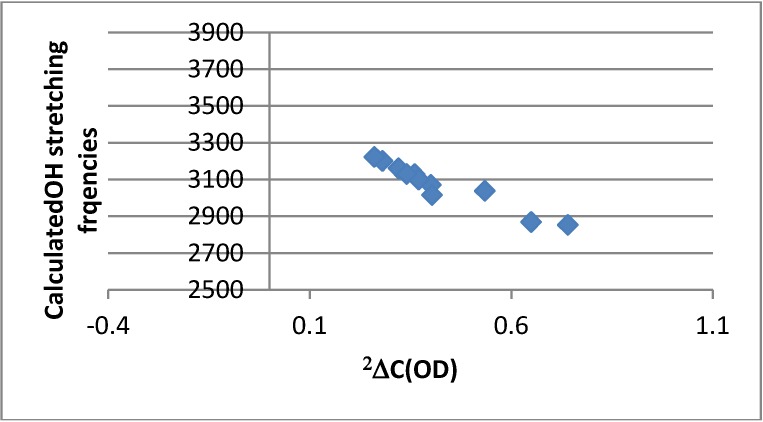
Plot of Calculated OH stretching frequencies *vs.* ^2^ΔC(OD).

OH stretching frequencies are calculated using the B3LYP functional and the 6-31G(d) basis set. They show a reasonable correlation both to OH chemical shifts (Figure 3) and two-bond deuterium isotope effects on ^13^C chemical shifts (Figure 8). In the latter case it is noticeable that the data plot for 4-nitro-HBQ in DMF-*d_7_* falls off the correlation line.

## 3. Discussion

The OH chemical shifts of all studied compounds were found to be large and were exceptionally large for the 7,9-dinitro-HBQ in DMF-*d_7_*. In the latter case this is partly due to a solvent effect. However, as seen in Figure 4 this is most likely also due to a ring current contribution of approximately 1.1 ppm. Another contributing factor could be that nitrogen is an acceptor rather than oxygen. A comparison in this case could be an azo-group as acceptor. However, in this case the OH chemical shifts are not very large and a comparison of ^2^ΔC(OD) and the OH chemical shifts for e.g., 2,4-dihydroxyazobenzene shows the opposite trend [25].

It is also remarkable that the correlation between the calculated OH nuclear shielding and the observed OH chemical shifts show that the OH nuclear shieldings are calculated too large (see Figure 6b). ^1^H nuclear shieldings and related to that ^1^H chemical shifts are normally calculated quite well using the GIAO approach [26,27]. The finding that this is not the case for the OH protons of this study suggests that the OH bond lengths are calculated too short (see below).

From the calculations of changes in nuclear shielding it is found that the change in OH nuclear shieldings is ~45 ppm/Å (see Experimental). From the plot of chemical shifts *vs.* nuclear shieldings it is seen that the OH nuclear shielding is calculated too high (Figure 6b). For the 9-dodecanoyl-HBQ the difference can be estimated as 2 ppm (see Figure 6b). Using the above figure of ~45 ppm/Å this translates into a bond length difference of 0.044 Å. The obtained OH bond length obtained for HBQ itself is much shorter than measured for the X-ray structure at room temperature [28], but larger than those obtained by using a higher basis set, B3LYP/6-311++G(d,p) [9].

From the slopes found by plotting observed deuterium isotope effects on ^13^C nuclear shieldings *vs.* calculated change in nuclear shielding with fixed OH bond length of 0.01 Å (Figure 7), it is seen that the slope increases gradually with the electron withdrawing power of the substituents. This means that the OH bond length changes in the same direction upon deuteriation. The larger change is clearly a sign of a more asymmetric hydrogen bond potential. This finding is supported by longer OH bond lengths and shorter O…N distances (see Table 4). These trends are also very clearly reflected in the ^2^ΔC(OD) isotope effects.

**Table 4:** Calculated OH bond lengths and O…N distances in Å.

Compounds	R_O…N_	R_OH_ (B3LYP)
HBQ	2.618	0.997
7-Dodecanoyl-HBQ	2.574	1.004
7-Formyl-HBQ	2.585	1.004
7-Nitro-HBQ	2.570	1.005
7-Azo-HBQ	2.597	1.002
9-Dodecanoyl-HBQ	2.588	1.000
7-Nitro-9-dodecanoyl-HBQ	2.533	1.014
7-Dodecanoyl-9-nitro-HBQ	2.537	1.014
4-Morpholino-HBQ	2.604	0.999
7,9-Dinitro-HBQ (NH-form)	2.478	1.079 (NH)

Changes in nuclear shieldings are calculated in the 9-dodecanoyl-HBQ. This demonstrates first of all that a large negative isotope effect is to be expected at the C=O carbon, as well as an effect at the CH_2_ group next to the carbonyl group. None of these effects are seen in the experimental spectra. Secondly, a very small effect is predicted at C-2 showing that the effect observed at this carbon is predominantly due to a transmission via the hydrogen bond between the OH group and the nitrogen.

An interesting finding is the ^2^ΔC(OD) of 4-nitro-HBQ. As seen from Figure 8 this is clearly too large. ^2^ΔC(OD) is measured in DMF-*d_7_* in contrast to most of the other compounds which are measured in CDCl_3_. Furthermore, deuteriation in DMF-*d_7_* is done by addition of an appropriate amount of heavy water. DMF-*d_7_* and/or water apparently lead to a larger deuterium isotope effect. This will also explain that the data points for 4-nitro-HBQ in Figure 2, Figure 8 is fall off the correlation lines.

In the 7,9-dinitro-HBQ a coupling is seen from the XH proton to H-2. This coupling is absent in the deuteriated sample. The magnitude of this coupling, 4 Hz, is rather unusual and is not seen in any of the monosubstituted cases. The XH chemical shift is very high, 20.2 ppm in DMF-*d_7_*. A correlation between observed ^13^C-NMR chemical shifts and calculated nuclear shieldings showed the best correlation coefficient for the NH form rather than for the OH form or for a tautomeric equilibrium. From the deuterium isotope effects on ^13^C chemical shifts (Figure 1) the effects are seen to be very different from those of the OH-forms again supporting the NH form. A reasonable fit is found between calculated changes in nuclear shieldings as given in Table 3 and the observed isotope effects (Figure 1). Again the solvent seems to be the driving force.

## 4. Experimental

### 4.1. General

All reagents and solvents were purchased from Sigma-Aldrich Chemical Co. (Brøndby, Denmark) and used without further purification. HBQ was purchased from TCI (Haven, Belgium). Fluka (Buchs, Switzerland) silicagel/TLC-cards 60778 with fluorescence indicator 254 nm were used for TLC chromatography. Fluka silicagel 60 (0.040–0.063) was used for flash chromatography purification of the products. Melting points were determined on a Gallenkamp apparatus and are uncorrected. The NMR spectra were recorded on a Varian Inova 600 or a Varian Mercury 300 NMR spectrometer in either CDCl_3_ or DMF-*d_7_*, using tetramethylsilane (TMS) as an internal standard. The mass spectra were measured on a LCQ-Deca ion trap instrument from Thermo-Finnigan, equipped with an atmospheric pressure chemical ionization interface (APCI) running in both negative and positive mode using the infusion technique.

The syntheses of 4-tosyl-HBQ and 4-morpholino-HBQ were previously described in Ref. [22]. 

### 4.2. Compounds

*7-Nitro-10-hydroxybenzo[h]quinoline*. To a solution of 10-hydroxybenzo[*h*]quinoline (0.67 g; 3.44 mmol) in acetic acid (15 mL), 65% HNO_3_ (1.5 mL) was added dropwise via cannula with vigorous stirring. The temperature was maintained at 15–20 °C and stirring continued for 2 h. The mixture was poured into water (150 mL), and left stirred overnight. The brown solid was filtered, washed with water until acid free. The solid was digested with hot ethanol and filtered, washed with ethanol and dried in an oven at 100 °C to give a light amber solid 0.64 g, mp. 198–200 °C, APCI-MS, 239.21 (M-1). For ^1^H and ^13^C-NMR data, see Table 1, Table 2. For a different approach see Ref. [22].

*7,9-Dinitro-10-hydroxybenzo[h]quinoline*. 65% HNO_3_ (5 mL) was charged into a 10 mL round bottom flask equipped with a magnetic stir bar. The acid was cooled to 0 °C (ice-bath) and 10-hydroxybenzo[*h*]quinoline (0.67 g; 3.44 mmol) was added in portions (over 30 min.). After complete addition, the clear orange solution was left stirring at 0 °C for 15 min. and then at 40 °C for 1.5 h. The mixture was cooled and poured into crushed ice with stirring. The yellow solid was collected by filtration washed with water until acid free and then washed with hot ethanol and dried in oven at 100 °C to give a yellow solid 0.61 g, mp. 260–262 °C, APCI-MS, 284.26 (M-1). For ^1^H and ^13^C-NMR data, see Table 1a, Table 2.

*7-Dodecanoyl-10-hydroxybenzo[h]quinoline and 9-dodecanoyl-10-hydroxybenzo[h]quinoline*. To a solution of 10-hydroxybenzo[*h*]quinoline (0.67 g; 3.44 mmol) in dry 1,2-dichloroethane (20 mL) was added lauroyl chloride (0.90 g; 4.12 mmol) dissolved in dry 1,2-dichloroethane (5 mL) at room temperature. The mixture was stirred for 30 min. and then anhydrous aluminum chloride (1.15 g; 8.61 mmol) was added in small portions during 30 min. After complete addition the mixture was stirred at room temperature for 30 min, and at 70 °C for 2 h. The dark brown mixture was cooled to 25 °C and carefully poured into a mixture of crushed ice and conc. hydrochloric acid with stirring and adjusted to pH 6.0 with 0.5 M NaOH and then extracted with chloroform (3 × 50 mL), washed with water (3 × 100 mL), brine and finally dried over anhydrous MgSO_4_, filtered and the solvent evaporated in vacuum to obtain a brown waxy crude product. The product mixture was purified by column chromatography on silica gel, using hexane-ethyl acetate (3:1 v/v) as eluent. The early fraction was the 9-isomer obtained as a light brown waxy material, 0.18 g. APCI-MS, 376.51 (M-1). For ^1^H and ^13^C-NMR data, see Table 1, Table 2. The later fraction was the 7-isomer obtained as a light amber solid 0.24 g, mp. 92–93 °C, APCI-MS, 376.48 (M-1). For ^1^H and ^13^C-NMR data, see Table 1, Table 2.

*7-Azobenzene-10-hydroxybenzo[h]quinoline*. Aniline (0.163 g; 1.79 mmol) was dissolved in a mixture of distilled water (5 mL) containing conc. HCl (0.45 mL) and cooled in ice bath. A solution of sodium nitrite (0.124 g; 1.79 mmol) in water (5 mL) was cooled to 0 °C and added slowly to the aniline solution. The mixture was left stirring at 0 °C for 15 min. The diazotized solution was added slowly to a solution of 10-hydroxybenzo[*h*]quinoline (0.349 g; 1.79 mmol) dissolved in a mixture of dichloromethane (5 mL),ethanol (5 mL) and KOH (0.103 g; 1.79 mmol) at 0 °C. After complete addition the mixture turned to red-orange color and left stirred at 0 °C for 45 min. The mixture was extracted with dichloromethane (3 × 30 mL), washed with water, brine and finally dried over anhydrous MgSO_4_. Evaporation of solvent afforded a red-orange solid. The crude product was purified by column chromatography on silica gel, using dichloromethane as eluent. The early fraction was the unreacted 10- hydroxybenzo[*h*]quinoline and the latter fraction was the 7-azo compounds obtained as orange solid 92.5 mg, mp. 158–159 °C, APCI-MS, 298.17 (M-1). For ^1^H and ^13^C-NMR data, see Table 1, Table 2.

*7-Formyl-10-hydroxybenzo[h]quinoline*. 10-Hydroxybenzo[*h*]quinoline (0.67 g; 3.44 mmol), chloroform (3 mL), ethanol (3 mL), NaOH (1.34 g, dissolved in 4 mL water) and tetraethylammonium bromide (20 mg) was charged into a steel cylinder. The cylinder was flushed with nitrogen, closed and heated in oil bath at 110 °C for 4 days. The cylinder was cooled and then opened carefully. The dark mixture was transferred to a flask with the aid of dichloromethane and water. The organic solvent was evaporated under reduced pressure and the dark residue was diluted with water (10 mL) and the pH was adjusted by 0.1 N HCl to 5.8. The mixture was extracted with dichloromethane (4 × 30 mL), washed with water and dried over anhydrous MgSO_4_. Evaporation of solvent afford dark brown solid residue. The product was purified by column on silica gel, using dichloromethane-ethyl acetate (10:2) as eluent. The early fraction was the unreacted 10-hydroxybenzo[*h*]quinoline 0.395 g. The later fraction was the 7-formyl isomer, which after solvent evaporation in vacuum give a light amber solid, 0.126 g, m.p. 177–179 °C, APCI-MS, 222.23 (M-1). For ^1^H and ^13^C-NMR data, see Table 1, Table 2.

*7-Nitro-9-dodecanoyl-10-hydroxybenzo[h]quinoline and 9-nitro-7-dodecanoyl-10-hydroxybenzo[h]quinoline*. A crude mixture sample of 7-dodecanoyl-10-hydroxybenzo[*h*]quinoline and 9-dodecanoyl-10-hydroxybenzo[*h*]quinoline (0.30 g) was dissolved in a mixture of acetonitrile (10 mL) and glacial acetic acid (10 mL), followed by addition of tetrabutylammonium bromide (150 mg). The mixture was stirred at room temperature for 10 min. and then 65% HNO3 was added slowly. After complete addition, the mixture was heated to 60 °C for 3 h with stirring. The mixture was left in cold place at 2.0 °C overnight, an orange solid was crystallized, filtered and washed with a little methanol and dried in vacuum at room temperature. The crud mixture was purified by column on silica gel, using hexane-ethyl acetate (4:1) as eluent. The early fraction was 7-dodecanoyl-9-nitro-isomer obtained as an orange solid, 86 mg. APCI-MS, 421.52 (M-1). For ^1^H and ^13^C-NMR data, see Table 1, Table 2. The later fraction was the 9-dodecanoyl-7-nitro isomer obtained as amber solid 72 mg. APCI-MS, 421.52 (M-1). For ^1^H and ^13^C-NMR data, see Table 1, Table 2.

### 4.3. Theoretical Calculations

The DFT calculations were performed with the Gaussian 09 package [29] and the molecular geometries were fully optimized using the B3LYP variant of the density functional theory (DFT) [30,31] with the 6-31G (d) basis set. The NMR nuclear shieldings were calculated with the same level of theory and basis set using the GIAO method [26,27].

## 5. Conclusions

Two-bond isotope effects on ^13^C-NMR chemical shifts are rather large in HBQs bearing electron withdrawing substituents in the 7 and 9 positions. This finding clearly signals a very asymmetric hydrogen bond. However, for HBQ itself, the hydrogen bond is not that strong or asymmetric. Using OH chemical shifts to judge the hydrogen bond strengths in these compounds is dangerous due to the large ring current effects seen in these compounds. Calculation of changes in nuclear shieldings and comparison with measured deuterium isotope effects on ^13^C chemical shifts is clearly a way to learn about the asymmetry of the hydrogen bond. Calculated OH nuclear shieldings are too large. This is also related to too short OH bond lengths, but a comparison of measured OH chemical shifts and calculated OH nuclear shieldings can lead to more realistic OH bond lengths.
